# Supplementary material for: Exploring barriers to the adoption and utilization of improved latrine facilities in rural Ethiopia: An Integrated Behavioral Model for Water, Sanitation and Hygiene (IBM-WASH) approach
Source: PLoS One. 2021 Jan 11;16(1):e0245289. doi: 10.1371/journal.pone.0245289 (PMC7799797; doi:10.1371/journal.pone.0245289)
Supplement: S1 File — (DOCX) [file pone.0245289.s001.docx]

**Topic Guide for Focus Group Discussions and In-depth Interviews**

Barriers to the Adoption and Utilization of Improved Sanitation Facilities in rural Ethiopia: An Integrated Behavioral Model for Water, Sanitation and Hygiene (IBM-WASH) Approach

Note: This topic guide is indicative

**Introduction:**

- Thank you for participating
- Self-introduction
- Introduce the study
- Talk about key points:
- duration of the interview/discussion
- no correct or incorrect answers
- voluntary participation, rights to withdraw
- Confidentiality and anonymity, how to disclose findings
- Questions?
- Ready to continue? Sign a form of consent
- **START RECORDING**

| **No** | **Questions/Topics** | **Probes** |
| --- | --- | --- |
| 1 | Respondent profile  To begin with, tell me about yourself  How long have you lived here? |  |
| 2 | How would you describe the general defecation habits of this community? | - What are your perceptions, attitude and norms about latrine construction and use in this community? - What is the general level of latrine construction and use in this community? |
| 3 | Have people in your community always had the same ideas about latrines? How have they changed or how have they remained the same over time? |  |
| 4 | What are the general characteristics of people who own and use latrines in this community? |  |
| 5 | In your community are there people who have latrines and do not use them? What could be the reasons for non-use of the latrines? |  |
| 6 | Are there people in your community who do not have latrines? What could be the main reasons for this? |  |
| 7 | Do you think people in this community have the capacity necessary to construct latrine facilities? (Skills, ability, materials, funds etc) |  |
| 8 | Who are the main people who promote latrine construction and use in this community and in what way? Whose responsibility do you think it is to improve access to latrines in your community? |  |
| 9 | What are your perceptions about handling children’s feces? |  |
| 10 | Looking to the future…   - Tell me about how you think improved latrine adoption and utilization can be improved |  |

**Closing**

- Anything to add? Anything that you feel is missing?
- Any questions?
- Copy of transcript?
- Summary of results from the study?
- Thank you very much!
