# Supplementary material for: Exploring barriers to the adoption and utilization of improved latrine facilities in rural Ethiopia: An Integrated Behavioral Model for Water, Sanitation and Hygiene (IBM-WASH) approach
Source: PLoS One. 2021 Jan 11;16(1):e0245289. doi: 10.1371/journal.pone.0245289 (PMC7799797; doi:10.1371/journal.pone.0245289)
Supplement: Attachment — Submitted filename: Response to reviwers.docx [file pone.0245289.s003.docx]

**Authors’ Response to the Academic Editor**

**Dear: Dr. Susan Horton, Academic Editor**

We thank you for your thorough reading and constructive criticism of our manuscript and for the opportunity to revise and resubmit. We are pleased to submit the revised research article entitled “Exploring Barriers to the Adoption and Utilization of Improved Latrine Facilities in rural Ethiopia: An Integrated Behavioral Model for Water, Sanitation and Hygiene (IBM-WASH) Approach” for your consideration in *PLOS ONE*. On the following page, you will find our response to your comments. On behalf of my co-author, I thank you again for your consideration of this resubmission. We appreciate your time and look forward to your response.

Sincerely,

Aiggan Tamene

[Corresponding author]

**Point by point response to the Editor’s comments**

We would like to thank you for your time and effort made to review our manuscript in detail. We believe that the comments have identified important areas that required improvement. After completion of the suggested edits, the revised manuscript has benefitted from an improvement in the overall presentation and clarity. We have used Microsoft review track change in the document to indicate any changes in word, phrase, or sentence.

**Comment:** Thank you for responding appropriately to the reviewers' comments. I would ask you to make one small additional further change, namely, just before Table 1, include a couple of sentences explaining how the participants were recruited for the two FGD.

**Response -** As the esteemed Editor rightly pointed out, an explanation of how the study participants were recruited was missing from the manuscript. We apologize for not addressing it after the first comment. We misunderstood the Editor’s comments and only added the eligibility criteria used in the present study. We want to emphasize that it was not the authors’ intention to not address such a critical revision to our manuscript. We used purposive sampling using local contacts as we reached a conclusion that random sampling when selecting FGD participants. Might compromise the findings as the groups need to be homogenous and all individual members need to share the characteristic relevant to our information needs in order for us to get the most out of the discussion

We hope the esteemed Editor can see that the revised manuscript has tried to accommodate the much deserved constructive criticisms to the fullest extent of our abilities.

Best Regards,

Thank you!
